# Contribution of brain pericytes in blood–brain barrier formation and maintenance: a transcriptomic study of cocultured human endothelial cells derived from hematopoietic stem cells

**DOI:** 10.1186/s12987-020-00208-1

**Published:** 2020-07-28

**Authors:** Marjolein Heymans, Ricardo Figueiredo, Lucie Dehouck, David Francisco, Yasuteru Sano, Fumitaka Shimizu, Takashi Kanda, Rémy Bruggmann, Britta Engelhardt, Peter Winter, Fabien Gosselet, Maxime Culot

**Affiliations:** 1 Laboratoire de la Barrière Hémato-Encéphalique (LBHE), Univ. Artois, UR 2465, 62300 Lens, France; 2 GenXPro GmbH, Frankfurt, Germany; 3 Johann Wolfgang Goethe University Frankfurt, Frankfurt, Germany; 4 Interfaculty Bioinformatics Unit and Swiss, Institute of Bioinformatics, University of Bern, Bern, Switzerland; 5 Department of Neurology and Clinical Neuroscience, Graduate School of Medicine, Yamaguchi University, Ube, Japan; 6 Theodor Kocher Institute, University of Bern, Bern, Switzerland

**Keywords:** Blood–brain barrier_1_, Transcriptome_2_, BBB formation_3_, In vitro_4_, Central nervous system_5_, Brain endothelial cells_6_, Human hematopoietic stem cells_7_, Brain pericytes_8_

## Abstract

Formation, maintenance, and repair of the blood–brain barrier (BBB) are critical for central nervous system homeostasis. The interaction of endothelial cells (ECs) with brain pericytes is known to induce BBB characteristics in brain ECs during embryogenesis and can be used to differentiate human ECs from stem cell source in in vitro BBB models. However, the molecular events involved in BBB maturation are not fully understood. To this end, human ECs derived from hematopoietic stem cells were cultivated with either primary bovine or cell line-derived human brain pericytes to induce BBB formation. Subsequently, the transcriptomic profiles of solocultured vs. cocultured ECs were analysed over time by Massive Analysis of cDNA Ends (MACE) technology. This RNA sequencing method is a 3′-end targeted, tag-based, reduced representation transcriptome profiling technique, that can reliably quantify all polyadenylated transcripts including those with low expression. By analysing the generated transcriptomic profiles, we can explore the molecular processes responsible for the functional changes observed in ECs in coculture with brain pericytes (e.g. barrier tightening, changes in the expression of transporters and receptors). Our results identified several up- and downregulated genes and signaling pathways that provide a valuable data source to further delineate complex molecular processes that are involved in BBB formation and BBB maintenance. In addition, this data provides a source to identify novel targets for central nervous system drug delivery strategies.

## Background

Brain capillary endothelial cells (ECs) display unique characteristics when compared to ECs from peripheral vasculature, e.g. tight junctions, low pinocytic activity, expression of metabolic enzymes, transporters, receptors and efflux pumps [24]. These characteristics are known to be the blood-brain barrier (BBB) phenotype which constitute the BBB [2, 11]. The BBB is the interface between the systemic circulation and the central nervous system and is essential to maintain brain homeostasis, thereby restricting the entry of many pathogens, toxins and compounds into the brain [1]. Several of these brain capillary EC characteristics mentioned above are demonstrated not to be intrinsic to brain ECs, however, they result from the regulation of cellular and non-cellular factors produced by different cell types of the neurovascular unit (NVU), e.g. astrocytes, pericytes, neurons, neuroglia and peripheral immune cells [9, 11, 30]. The specific crosstalk between brain ECs and brain pericytes is known to induce BBB characteristics (e.g. expression and functionality of tight junction proteins, decreasing leukocyte adhesion molecule expression, decreasing transcytosis and induction of the basement membrane) in ECs during embryogenesis in vivo [11, 30]. Pericytes are a type of vascular cells embedded in the basement membrane, thereby they wrap the cerebral capillary walls, with a pericyte coverage being the highest in neural tissue [30]. The latter implicates the importance of pericytes for BBB functioning, which is as well indicated by studies that relate pericytes to barrier function and regulation of inflammatory responses [9, 22]. The pericyte-brain EC interaction is also used to differentiate ECs from stem cell source to human brain-like ECs which are used in in vitro BBB models [7, 21, 23, 36]. These in vitro models should display barrier tightening, i.e. induced by coculture, in order to be of use for pharmaceutical screening. However, the underlying molecular events involved in development, maturation and maintenance of BBB features, are not fully understood and difficult to study in vivo, especially in humans. In particular, the BBB regulation related to the communication between pericytes and brain ECs remains largely unknown [5, 15, 22].

In the present study, we make use of a human in vitro BBB model developed by Cecchelli et al. [7] consisting in ECs derived from hematopoietic stem cells which are co-cultivated with brain pericytes. After 5 days of coculture with brain pericytes, the ECs were shown to display features of the BBB which were absent when the cells were cultivated alone: the co-cultivated ECs display a continuous expression of ZO-1, occludin, JAM-A, claudin-1 and claudin-5 at cell–cell contacts resulting in a lower permeability to non-permeant marker than when the cells were solocultivated. These cocultivated ECs also express several transporters typically observed in brain endothelium in vivo (e.g. ABCB1 and ABCG2) [7].

To study the molecular processes responsible for the observed changes in ECs (i.e. barrier tightening, changes in the expression of transporters and receptors) in coculture with brain pericytes of ECs derived from hematopoietic stem cells cultivated with brain pericytes from either primary bovine or cell line human origin in a Transwell system using the Massive Analysis of cDNA Ends (MACE) technology. This RNA sequencing method is a 3′-end targeted, tag-based, reduced representation transcriptome profiling technique, that can reliably quantify all polyadenylated transcripts including those with low expression. By analysing the generated transcriptomic profiles, we can explore the molecular processes responsible for the functional changes observed in ECs in coculture with brain pericytes.

To specifically focus on the pericyte-EC interaction, we decomposed the model in either solo- or cocultured ECs. Human ECs were cultivated (in a non-contact set-up) with either human pericytes (CHP) or with bovine brain pericytes (CBP). In both coculture conditions, the ECs display BBB characteristics like restrictive tight junctions, low paracellular permeability to integrity markers and functional expression of polarized uptake and efflux transporters [7]. We subsequently compared the transcriptomic profile of cocultured ECs to the transcriptomic profile of solocultured ECs to delineate the transcriptional changes occurring in the ECs during barrier establishment. Besides the transcriptomic data, BBB functions were assessed by drug accumulation and permeability studies to preliminary validate the physiological relevance of the used in vitro model.

Transcriptomic profiling was done using high-throughput mRNA sequencing in combination with the digital gene expression profiling technique of GenXPro (Frankfurt am Main, Germany), the MACE technology. MACE performs gene expression profiling by sequencing part of the 3′-end of mRNA transcripts. Since each sequenced read represents one single mRNA molecule, the MACE technique can accurately quantify polyadenylated transcripts using a considerably lower sequencing depth than that of standard RNA-sequencing protocols, for which the number of fragments per transcript depends on the length of the transcript.

Our results provide a transcriptomic landscape of human brain-like ECs in solo- or coculture with brain pericytes that was used to identify interesting gene profiles over time, soloculture enriched transcripts, coculture enriched transcripts, etc., which might prove to be valuable in the further delineation of complex molecular processes involved in BBB formation and regulation. The transcriptomic profile could also be used as a source for novel targets for central nervous system drug delivery strategies.

## Materials and methods

### Compounds

The compounds lucifer yellow (LY; Mw = 457.25 g mol^−1^), rhodamine 123 (R123; Mw = 380.82 g mol^−1^) and elacridar (GF; Mw = 563.65 g mol^−1^) and other materials like bovine serum albumin and dimethyl sulfoxide, were purchased from Sigma-Aldrich (St. Quentin Fallavier, France).

All powdered compounds were dissolved in dimethyl sulfoxide or Krebs-Ringer HEPES (RH) buffer (NaCl 150 mM, KCl 5.2 mM, CaCl_2_ 2.2 mM, MgCl_2_ 0.2 mM, NaHCO_3_ 6 mM, glucose 2.8 mM, HEPES 5 mM, sterile water for injection—pH: 7,4). The source and origin of all other materials used in this study are detailed throughout the methodology.

### Cell culture

#### Soloculture of hematopoietic stem cell-derived endothelial cells

The human in vitro BBB model used in this study was modified from the coculture model of Cecchelli et al. [7]. In brief, hematopoietic stem cell-derived ECs were isolated according to the method described in Cecchelli et al. [7]. Vials of frozen ECs (1 × 10^6^ cells) were rapidly thawed and seeded in gelatin-coated (type A from porcine skin) (Sigma-Aldrich) 100-mm Petri dishes (Costar, Corning Incorporated, NY, USA) containing complete medium for ECs i.e. endothelial cell medium (Sigma-Aldrich), supplemented with 5% fetal calf serum (Integro), 1% endothelial cell growth supplement (Sigma-Aldrich) and 0.5% gentamicin (Biochrom AG, Berlin, Germany). Two days after defrosting, around 5.0 × 10^6^ cells were present and ECs were trypsinized with trypsin/ethylenediaminetetraacetic acid (0.05%/0.02% in phosphate buffered saline-calcium and magnesium free (Biochrom AG) and seeded on a semi-permeable Transwell insert (0.4 mm, 12-well system, Costar, Corning Incorporated) coated with Matrigel (growth factor reduced BD Matrigel Matrix, BD Biosciences), at a concentration of 16.0 × 10^4^ cells/mL. Cells were cultivated at 37 °C in a humified atmosphere at 5% CO_2_/95% air for a total of 7 days and medium was changed every 2 days. All sera were heat-inactivated before use.

#### Coculture of stem cell-derived ECs with brain pericytes

Primary bovine brain pericytes were isolated from the brain of freshly killed cows obtained from the slaughterhouse of Douai, France according to the method described by Vandenhaute et al. [36]. Vials of frozen primary bovine brain pericytes (passage ≤ 3; 1.0 × 10^6^ cells) were rapidly thawed and seeded in gelatin-coated 100-mm Petri dishes containing complete medium for bovine pericytes (Dulbecco’s modified eagle’s medium (Gibco, Thermo Fisher Scientific, Villebon-sur-Yvette, France) supplemented with 20% fetal calf serum, 1% l-glutamine (Merck Chemicals, Darmstadt, Germany) and 0.5% gentamicin). After 2 days, bovine pericytes were trypsinized and seeded, at a concentration of 1.3 × 104 cells/cm^2^ on the bottom of gelatin-coated 12-well plates (Costar, Corning Incorporated).

The cell line of Human brain pericytes (hBPCT cell line) was provided by Yamaguchi University, Japan and derived from primary brain pericytes of a patient that died from a heart attack isolated and immortalized with retroviral vectors harboring a SV40 large T antigen gene according to the method described by Shimizu et al. [32]. Vials of frozen human brain pericyte between passage 15 and 25 (1.0 × 10^6^ cells) were rapidly thawed and seeded onto rat tail collagen (type I)-coated (BD Biosciences) 100-mm Petri dishes containing complete medium for human pericytes (Dulbecco’s modified eagle’s medium supplemented with 10% fetal calf serum, 1% l-glutamine and 1% penicillin–streptomycin (Sigma-Aldrich)). The rat tail collagen was prepared as described by Dehouck et al. [12]. After 2 days, human pericytes were trypsinized and seeded, at a concentration of 1.3 × 10^4^ cells/cm^2^ on the bottom of rat tail collagen type I-coated 12-well plates.

Bovine and human brain pericytes were thawed 2 days before starting the coculture with ECs. Both cocultures were initiated by inserting the Transwell membranes with attached ECs into the pericyte-containing 12-well plates and by changing medium to endothelial cell medium, resulting in a non-contact BBB in vitro model, as no physical interaction exist between the two cell types. Experiments were initiated at different time points (i.e. 0, 24, 48 and 96 h) starting from t0, as the moment of coculture initiation. Cocultures were cultivated at 37 °C in a humified atmosphere and 5% CO_2_. All sera were heat-inactivated before use.

### Drug accumulation and permeability studies

#### Permeability experiments

At the different time points after putting ECs in coculture (i.e. 0, 24, 48, 72, 96 and 120 h), permeability experiments were performed to assess the EC monolayer tightness according to the method described in Vandenhaute et al. [36]. In brief, permeability was assessed by calculating the permeability coefficient of a fluorescent integrity marker (i.e. LY). To initiate experiments, Transwell inserts containing confluent monolayers of ECs, were loaded with 0.5 mL donor solution (i.e. LY (50 mM) in RH buffer) and were subsequently placed in a new 12-well plate filled with preheated RH buffer (1.5 mL). Cells were subsequently incubated (37 °C, 5% CO_2_) for exactly 60 min after which aliquots were taken from the initial donor solution (C0) and from the donor and receiver solutions at the end of the experiment (De) and (Re). The fluorescence intensity, hence, concentration of LY, was determined by using a fluorescence multiwell plate reader (Synergy H1 multiplate reader, BioTek Instruments SAS, Colmar, France), using a LY filter pair of Ex (λ) 432; Em (λ) 538 nm. Experiments were done in triplicate (i.e. 3 inserts), hence a total of 3 inserts with (i.e. filter + cells) and 3 inserts without (i.e. only filter) cells were assessed per condition. Simultaneously, blank wells were prepared using the same solution to assess background values for subtraction from the measured values.

The permeability coefficient (Pe, in cm min^−1^) and clearance were calculated according to the clearance principle described by Siflinger-Birnboim et al. [33]. The clearance principle was used to obtain a concentration-independent transport parameter.

The cleared volume (CL, in mL) was calculated by dividing the diffused amount of compound in the receiver compartment (Ar) with the concentration of compound in the donor compartment (Cd) (Eq. 1).

1 $$Clearance \left( {CL, \;in \;{\text{m}}L} \right) = {\raise0.7ex\hbox{${A_{r} }$} \!\mathord{\left/ {\vphantom {{A_{r} } {C_{d} }}}\right.\kern-0pt} \!\lower0.7ex\hbox{${C_{d} }$}}$$

The average cumulative CL was subsequently plotted over time and the slope was estimated by linear regression analysis. This resulted in the permeability-surface area product (PS, in mL min^−1^). To make a correction for permeability across cell-free inserts, the PS products was calculated for both cell-free inserts (i.e. PSf, filter) and inserts with cells (Eq. 2).

2 $$PS_{t} = PS_{f} + PS_{e}$$

The true or absolute Pe was then computed out of PSf and PSt (Eq. 3), normalized by the surface (S, in cm^2^) (Eq. 4) [7, 16, 36].

3 $$PSe^{ - 1} = PSt^{ - 1} - PSf^{ - 1}$$

4 $$Pe\; \left( {in \;{\text{cm}}\;{ \hbox{min} }^{ - 1} } \right) = \frac{PSe}{S}$$

The mass balance or recovery (in %) was determined to avoid deviating results due to a possible loss of the tracer by e.g. adsorption to plastics and non-specific binding to cells. The recovery was calculated by dividing the amount of recovered compound at the end of the experiment by the initial amount of tracer at t0. For Pe determination, a threshold recovery range was adopted between 80 and 120%.

#### Rhodamine accumulation studies

Drug accumulation assays were performed to evaluate functional activity of P-gp in ECs. The solo- and cocultured ECs were incubated for 2 h with R123 (5 mM) in RH buffer (supplemented with 0.1% bovine serum albumin) with or without GF (0.5 mM). After incubation, ECs were washed 3 times with ice-cold RH buffer and were subsequently lysed with lysis buffer (10× RIPA lysis buffer, Millipore Merck, Darmstadt, Germany). Fluorescence detection was performed with the fluorescence multiwell plate reader, using an R123 filter pair of Ex (λ) 501; Em (λ) 538 nm. Experiments were done at 37 °C in a humified atmosphere at 5% CO_2_.

### Statistical analysis

All results were expressed as means with standard deviation from three or more independent experiments. Statistical significance was assessed by the unpaired Student’s t-tests with two-tailed distribution, assuming equal standard deviation, or otherwise specified. A p-value < 0.05 was considered as significant (*p < 0.05; **p < 0.005, ***p < 0.001). All statistical analyses were performed using GraphPad Prism 7 for Mac OS X (GraphPad Software, San Diego, California, USA).

### RNA sequencing: Massive Analysis of cDNA Ends (MACE) technology

#### Total RNA isolation

RNA isolation was performed at GenXPro GmbH. Cell lysates were stored in liquid nitrogen before RNA isolation. Isolation of total RNA from ECs was performed using the ZR-Duet DNA/RNA MiniPrep Plus kit (Zymo Research, Irvine, CA, USA) according to manufacturer’s instructions. RNA samples were digested using DNase I and RNA integrity was accessed using automated capillary electrophoresis (RNA pico sensitivity assay, LabChip GX II Touch HT, Perkin Elmer, Villebon-sur-Yvette, France).

#### Generation of MACE libraries and RNA sequencing

We performed genome-wide gene expression profiling of solo- and cocultured ECs at 0, 24, 48 and 96 h after putting ECs in coculture (3 biological replicates each consisting of 3 technical replicates) using the MACE method to identify differentially expressed genes upon pericyte introduction. The biological replicates were defined as coming from different vials of frozen ECs. These replicates originate from the cord blood of 1 or 2 donors. For each biological replicate, we subsequently pooled 3 inserts (i.e. technical replicates). Hence, a total of 9 replicates was used.

Preparation of a next-generation sequencing library and subsequent RNA sequencing was performed at GenXPro GmbH. A number of 27 MACE libraries was constructed using the MACE-Seq kit v2.0 (GenXPro GmbH) according to the supplier’s protocol. MACE-sequencing is a 3′-end targeted, tag-based, reduced representation transcriptome profiling technique that can reliably quantify all polyadenylated transcripts.

In general, the procedure follows a modified protocol described in Nold-Petry et al. [29]. In brief, samples with 100 ng of DNase-treated RNA were used for library preparation. Synthesis of cDNA was performed by reverse transcription using oligo (dT) primers following fragmentation of cDNA to an average size of 200 bp using sonification (Bioruptor, Diagenode, Seraing, Belgium). DNA was quantified using a Qubit HS dsDNA assay (Thermo Fisher Scientific). cDNA fragments were ligated to DNA adapters containing TrueQuant unique molecular identifiers included in the kit. Library amplification was done using polymerase chain reaction, purified by solid phase reversible immobilization beads (Agencourt AMPure XP, Beckman Coulter, Brea, CA, USA) and subsequent sequencing was performed using a NextSeq platform (Illumina Inc., San Diego, CA, USA).

#### Bioinformatic analysis of MACE data

A total of approximately 391 million MACE reads was obtained across all libraries (Additional file 1). Polymerase chain reaction-duplicates were identified using the TrueQuant technology and subsequently removed from raw data. All remaining reads were further poly (A)-trimmed and low-quality reads were removed, after which clean reads were aligned to the human reference genome^1^ using the bowtie2 mapping tool. The latter resulted in a gene dataset with a total of 25,684 different genes. The gene count data was normalized to account for differences in library size and RNA composition bias by calculating the median of gene expression ratios using DESeq 2 R/Bioconductor package [27]. This resulted in a p-value and log2-fold change (log2FC) for every gene for 2 conditions. False discovery rate was estimated to account for multiple testing. During bioinformatic analysis, differentially expressed transcripts were identified using a combination of thresholds for p-value < 0.05 and |log2FC| > 1, as performed by Munji et al. [28]. During experimental design, these thresholds were considered to be correct to analyse even the most subtle changes in gene expression during the time course of the experiment, due to the characteristics of the experimental setup, biological questions and analysis. Additionally, the accurate quantification of mRNA transcripts using MACE sequencing allowed identification of differentially expressed genes using the combination of p-value and log2FC.

Differentially expressed genes were further categorized in solo- and coculture enriched genes. The ratio between the normalized expression of a specific gene in solocultured ECs and the normalized expression of the same gene in cocultured ECs resulted in soloculture enriched genes if the ratio exceeded 2, or otherwise specified. The ratio between the normalized expression of a specific gene in cocultured ECs and the normalized expression of the same gene in solocultured ECs resulted in cocultured enriched genes if the ratio exceeded 2. To obtain enriched genes, some thresholds were made to ensure to have taken into account only valuable genes (i.e. raw data count of the enriched condition > 20, exclusion of pseudogenes and non-coding genes).

Genes were further assigned to biological pathways to analyse signaling and metabolic pathways by using the Gene Ontology (GO) enrichment tool (GenXPro GmbH), the Ingenuity Pathway Analysis and the KOBAS web server. These functional enrichment tools were used to evaluate the functional properties of gene sets, thereby resulting in over- or underrepresented GO terms for a set of genes that were up- or downregulated in our comparisons. The used software consists of databases that classifies genes according to their roles in the cell, allowing to identify ‘pericyte or coculture enriched’ signaling pathways [18]. Statistical analysis of the GO enrichment analysis consisted of the Fisher’s exact test among transcripts that were differentially expressed at a p-value < 0.05.

#### Availability of data

The generated transcriptomic data for this study, including both the raw data and the counts matrix, has been deposited in the Gene Expression Omnibus (GEO) database with the ascension ID of GSE144474. The data will also be made available at the BBBHub (http://bbbhub.unibe.ch) upon launch.

## Results

### Influence of brain pericytes on functional barrier properties: Barrier tightness and efflux transporter functionality

The effect of brain pericytes on the barrier tightness and efflux transporter functionality in ECs was assessed by permeability studies to determine the tightness of the endothelial monolayer and by drug accumulation studies to assess functionality of ATP-binding cassette (ABC) efflux transporters e.g. *P*-glycoprotein (P-gp/ABCB1/MDR1) and breast cancer resistance protein (BCRP/ABCG2).

**Fig. 1:**
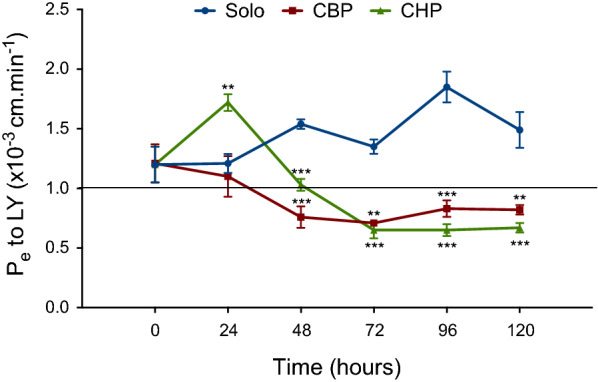
Endothelial permeability coefficient (P_e_) to LY (50 mM) over time of the endothelial cell monolayer in soloculture (solo, blue), in coculture with bovine pericytes (CBP, red) and in coculture with human pericytes (CHP, green). Data is shown as a mean (N = 3) ± standard deviation, statistics were done by a two-tailed unpaired t-test and a significantly different P_e_ to LY in CBP or CHP compared to soloculture is indicated by *p < 0.05, **p < 0.005, and ***p < 0.001

The ECs monolayer’s tightness was investigated by studying the Pe of the commonly used hydrophilic integrity marker, LY, across the endothelial cell monolayer. Both pericyte co-cultures (i.e. CBP and CHP) significantly decreased the permeability to LY over time (Fig. 1). The Pe to LY for solocultured ECs remained higher and relatively stable throughout the whole-time range, with an average over time of 1.36 ± 0.27 × 10^−3^ cm.min^−1^. The Pe to LY for the CBP and CHP decreased, when compared with the Pe of the solocultured ECs, and this by 37% (CBP) and 14% (CHP) after 48 h, by 31% (CBP) and 46% (CHP) after 96 h and by 32% (CBP) and 45% (CHP) after 120 h. The Pe to LY is 1.10 ± 0.17 × 10^−3^ cm min^−1^ and 1.72 ± 0.07 × 10^−3^ cm min^−1^ after 24 h and 0.82 ± 0.04 × 10^−3^ cm min^−1^ and 0.67 ± 0.04 × 10^−3^ cm min^−1^ after 120 h for the CBP and CHP respectively. These results demonstrate a common reduction of endothelial permeability over time for ECs cocultured with brain pericytes, which confirms the involvement of brain pericytes in regulating and/or inducing important BBB features. The latter has been shown by several other studies [8, 10, 11, 22].

**Fig. 2:**
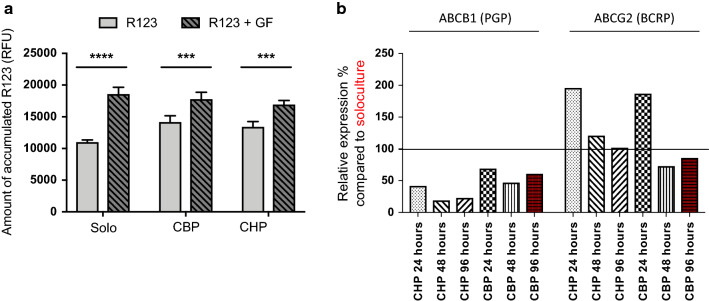
**a** Efflux pump activity measured by the intracellular accumulation of rhodamine 123 (R123) in absence (red) or presence (green, lines) of the inhibitor, elacridar (GF) in solocultured endothelial cells (Solo), or in cocultured endothelial cells with bovine pericytes (CBP) and cocultured endothelial cells with human pericytes (CHP). Data is shown as the mean amount intracellular accumulated R123 with standard deviation (in RFU) (N = 3). **b** Expression profile of ABC efflux transporters P-gp and BCRP in endothelial cells cocultured with human pericytes (CHP, green) or bovine pericytes (CBP, red). Expression is depicted as relative expression (in %) compared to the expression in solocultured endothelial cells at the corresponding time points

The functionality of the P-gp and BCRP efflux pumps was evaluated by a drug accumulation assay with P-gp and BCRP substrate R123. Cells were incubated with R123 in presence and absence of a P-gp and BCRP inhibitor, GF. Our results evidence the presence of functional efflux pumps in both solo- and cocultured ECs as demonstrated by an increased intracellular accumulation of R123 in presence of inhibitor (i.e. 43% and 42% for the CBP and CHP, respectively), compared to the baseline condition (i.e. depicting R123 accumulation in absence of GF) (Fig. 2a). Although not significantly different from the cocultured ECs, the difference between the intracellular accumulation of R123 in absence and in presence of inhibitor seems to be somewhat higher in the solocultured ECs. This is also reflected by the transcriptomic data (Fig. 2b) that shows an overall downregulation of P-GP and a decreasing expression of BCRP over time in cocultured ECs compared to solocultured ECs. Several studies show a pericyte-enhanced P-gp function or a higher P-GP expression in rodent brain vasculature compared to peripheral vasculature [10, 11, 15]. The latter is not reflected by our data.

### Influence of brain pericytes on the transcriptomic profile of brain-like endothelial cells

To identify the influence of brain pericytes on the transcriptomic expression profile of ECs, we utilized the MACE RNA sequencing technique. Therefore, we compared the gene expression of solocultured ECs to the gene expression of cocultured ECs.

#### Influence of pericytes on the global gene expression profile of brain-like endothelial cells

The MACE gene expression profiling of ECs in soloculture or in coculture with brain pericytes identified several clusters of gene responses: (i) up- or downregulated genes in cocultured ECs compared to solocultured ECs; (ii) up- or downregulated genes in ECs in the CBP compared to solocultured ECs; (iii) up- or downregulated genes in ECs in the CHP compared to solocultured ECs; and (iv) up- or downregulated genes at specific time points in cocultured ECs compared to solocultured ECs (Table 1).

**Table 1:** Summary of different clusters of gene responses

	Differentially expressed	Down-regulated	Up-regulated	Total regulated	Down-regulated	Up-regulated	Total regulated
	%	(p-value < 0.05; |log2FC| | > 1)			(p-value < 0.05; |log2FC| > 2)		
CBP vs. Solo (h)							
24	5.8	207	114	321 (1.2%)	49	26	75 (0.3%)
48	14.6	302	343	645 (2.5%)	70	109	179 (0.7%)
96	15.1	513	253	766 (3.0%)	148	72	210 (0.8%)
CHP vs. SOLO (h)							
24	20.4	414	534	948 (3.7%)	41	199	240 (0.9%)
48	16.1	297	561	858 (3.3%)	54	188	242 (0.9%)
96	18.7	644	591	1235 (4.8%)	114	260	374 (1.5%)

The percentage of differentially expressed transcripts (p < 0.05) is shown in the second column for every condition and is depicted as a percentage of the total number (i.e. 25 684) of mapped genes. Column 3 and 4 show differentially expressed transcripts in cocultured vs. solocultured endothelial cells characterized by a *|l*og2FC| > 1. Column 6 and 7 depict differentially expressed transcripts characterized by a |log2FC| > 2

**Fig. 3:**
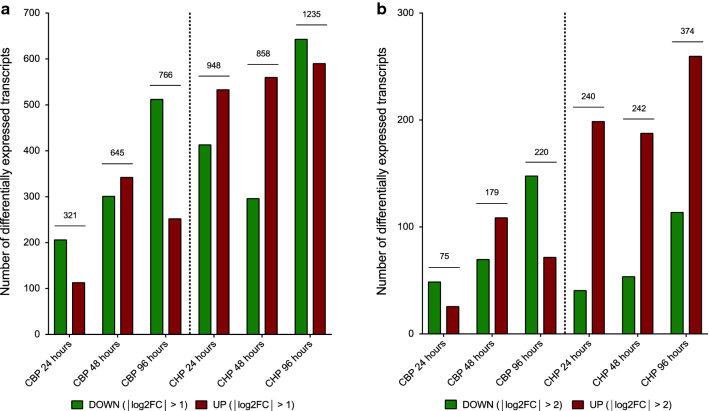
Number of differentially expressed transcripts for the cocultured vs. solocultured endothelial cells at different time points i.e. 24, 48 and 96 h. Coculture conditions were depicted as CBP for coculture with bovine pericytes and CHP for cocultures with human pericytes. Differentially expressed transcripts are characterized by (**a**) a |log2FC | > 1; and **b** a |log2FC| > 1 and categorized in up- (green)- and down- (red) regulated transcripts in cocultured endothelial cells compared with their expression in solocultured endothelial cells. The total number of transcripts in line with these statistics are depicted on top of the bars

Figure 3a shows the number of differentially expressed genes (|log2FC| > 1) for the comparison between cocultured ECs and solocultured ECs over time. This graph indicates an increase in differentially expressed genes over time for both cocultures, as well as it shows a higher number of differentially expressed genes in ECs from CHP compared with CBP. Figure 3b shows the number of differentially expressed genes (|log2FC| > 2) for the comparison between cocultured ECs and solocultured ECs over time. This graph shows (i) a higher number of differentially expressed genes in the comparison of soloculture vs. CHP, compared to the comparison of soloculture vs. CBP; (ii) an increased amount of differentially expressed genes over time for both the comparison of soloculture vs. coculture in general; and (iii) a clearly higher number of upregulated differentially expressed genes compared to downregulated differentially expressed genes for the comparison soloculture vs. CHP, which is not reflected in the comparison soloculture vs. CBP.

These results suggest that coculturing with human pericytes affects the gene expression profile more rapidly and slightly more than coculturing with bovine pericytes. However, the influence of pericytes is minor in both cases, as no more than 5% of the total number of genes is altered significantly upon coculturing. However, numerous genes are significantly affected (p-value < 0.05 and |log2FC| > 1) or |log2FC| > 2), but the change in expression levels is small, indicating a low responsiveness towards factors originating from pericytes.

#### Enriched gene expression

**Fig. 4:**
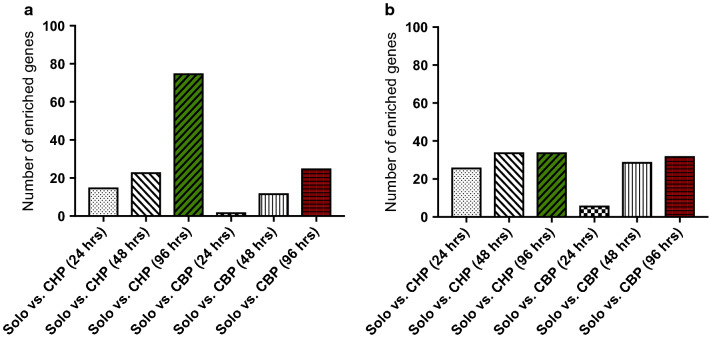
**a** Number of soloculture enriched genes (ratio ≥ 3) for solo- vs. coculture comparisons at different time points i.e. 24, 48 and 96 h. **b** Number of coculture enriched genes (ratio ≥ 3) for solo- vs. coculture comparisons. Coculture with bovine pericytes (CBP) depicted in red and coculture with human pericytes (CHP) depicted in green. The comparisons at 24 h were performed with a soloculture at 0 h, as no analysis was performed for a soloculture at 24 h

Differentially expressed genes were categorized in soloculture enriched and coculture enriched genes. Genes were identified as soloculture enriched when expressed at high levels in soloculture conditions and poorly or not expressed in coculture conditions. The opposite was true for coculture enriched genes. Our results indicate an increased number of soloculture enriched genes over time (ratio ≥ 3) (Fig. 4a), as well as an increased number of coculture enriched genes over time (ratio ≥ 3) (Fig. 4b) (i.e. for both the CHP and the CBP). Interestingly, the identified number of soloculture enriched genes in the comparison with the CHP shows to be threefold higher than for any other comparison.

A list of the ten of most enriched soloculture and coculture genes, for every comparison at each time point, was generated (Tables 2, 3). Within this list, genes that were identified to be enriched in both comparisons (i.e. soloculture vs. CHP and soloculture vs. CBP) are depicted in bold. These top regulated genes (Tables 2, 3) are partially validated by Ingenuity Pathway Analysis as several of the coculture enriched genes are found back in the list of top regulated genes from the Ingenuity Pathway Analysis (data not shown).

**Table 2:**
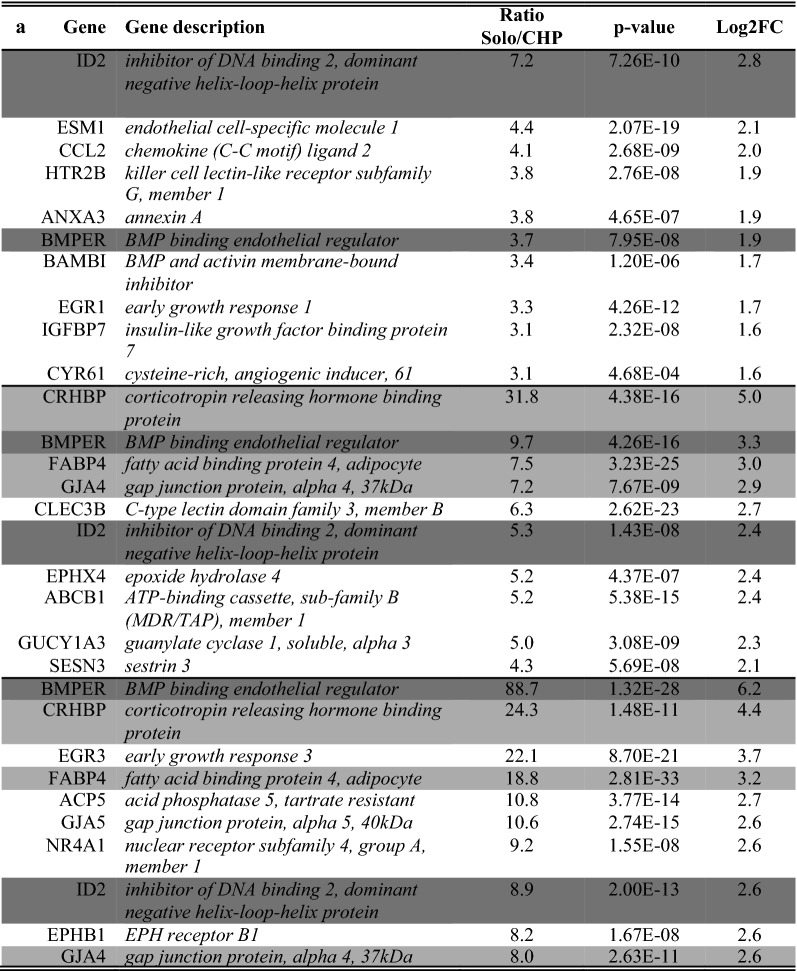

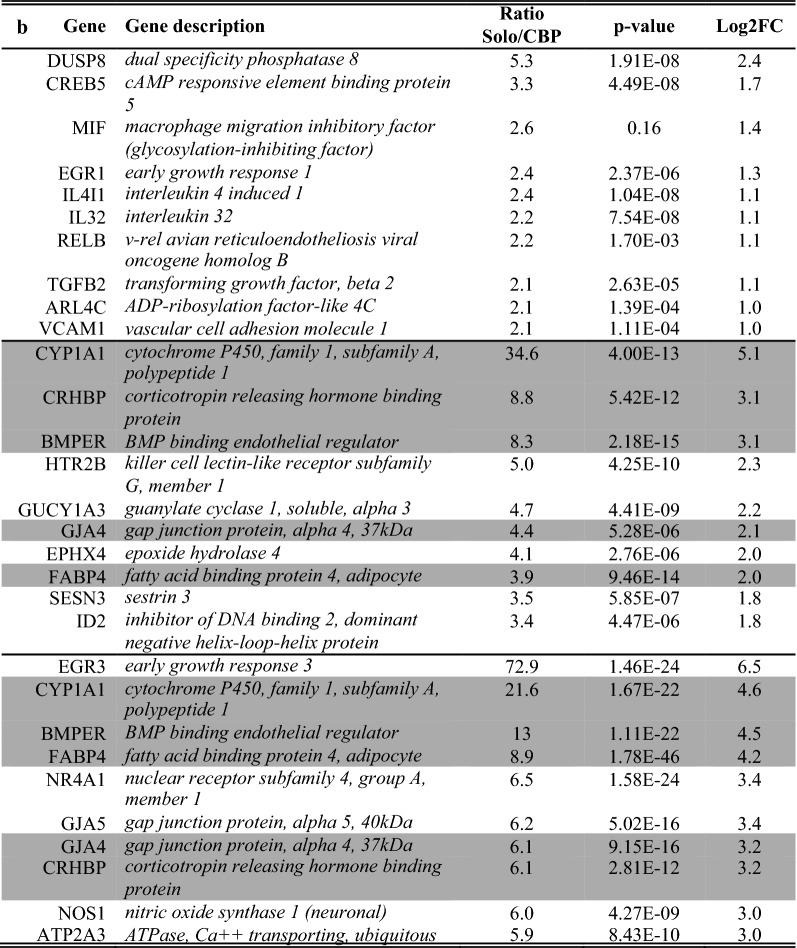
Top 10 of the most enriched soloculture genes at 24 h (top), 48 h (middle) and 96 h (bottom) for (A) the comparison of soloculture (Solo) vs. coculture with human pericytes (CHP); and for (B) the comparison of soloculture vs. coculture with bovine pericytes (CBP)

Shading color highlights soloculture enriched genes that are identified to be in the top 10 of two (light grey) or all time points (dark grey)

**Table 3:**
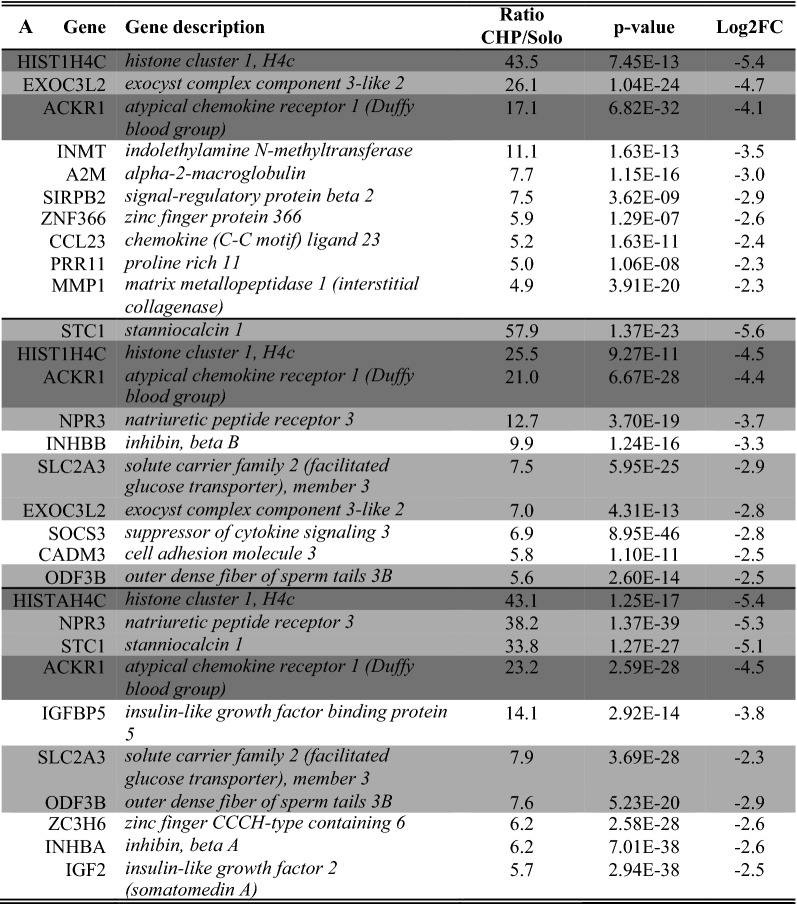

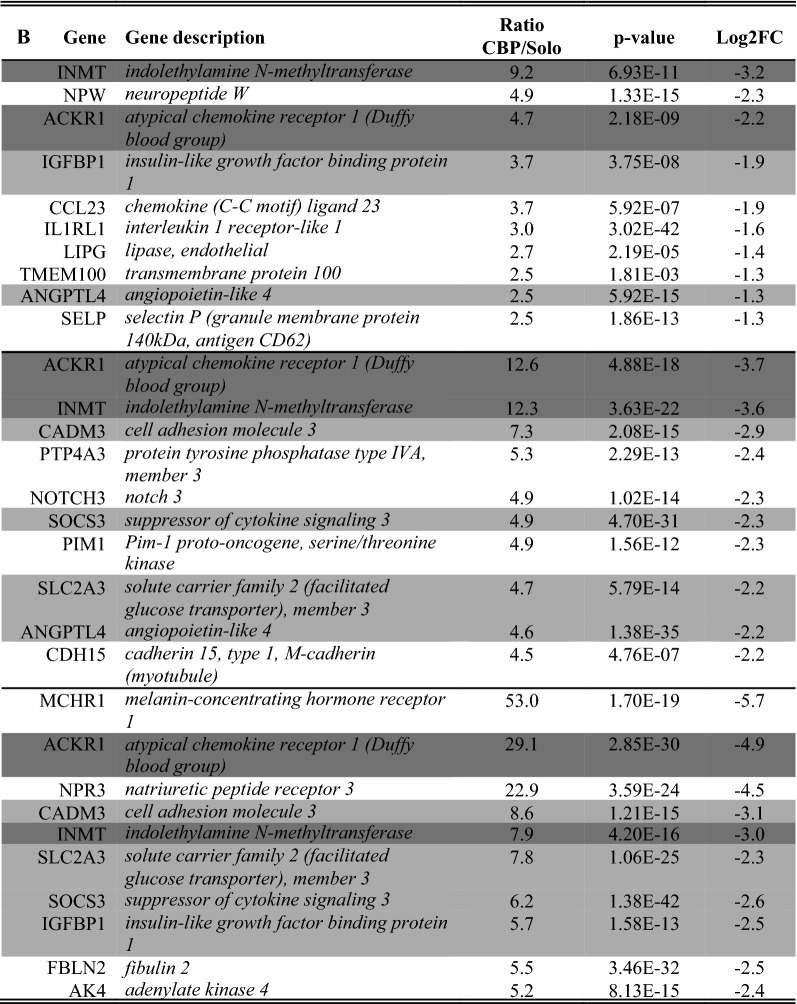
Top 10 of the most enriched coculture genes at 24 h (top), 48 h (middle) and 96 h (bottom) for (A) the comparison of soloculture (Solo) vs. coculture with human pericytes (CHP); and for (B) the comparison of soloculture vs. coculture with bovine pericytes (CBP)

Shading colour highlights coculture enriched genes that are identified to be in the top 10 of two (light grey) or all time points (dark grey)Shading colour highlights coculture enriched genes that are identified to be in the top 10 of two (light grey) or all time points (dark grey)

As overlapping genes between the two cocultures can be a first type of validation, a full comparison of overlapping genes in both comparisons was made. For this, we assessed the number of overlapping enriched genes in both comparisons (i.e. solo- vs. CHP and solo- vs. CBP). We only included those enriched genes that were characterized by a ratio ≥ 2 (Table 4 and 5). This assessment indicated that both soloculture and coculture overlapping enriched genes increase in number over time.

**Table 4:**
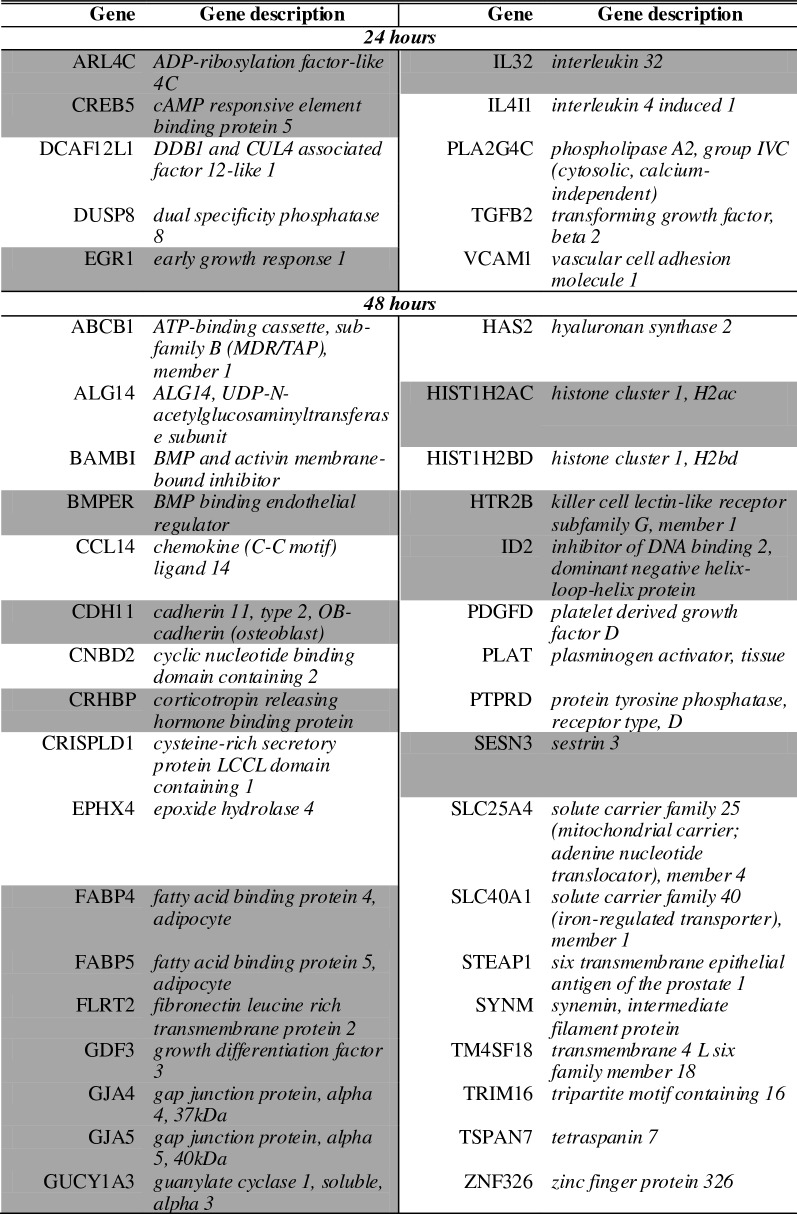

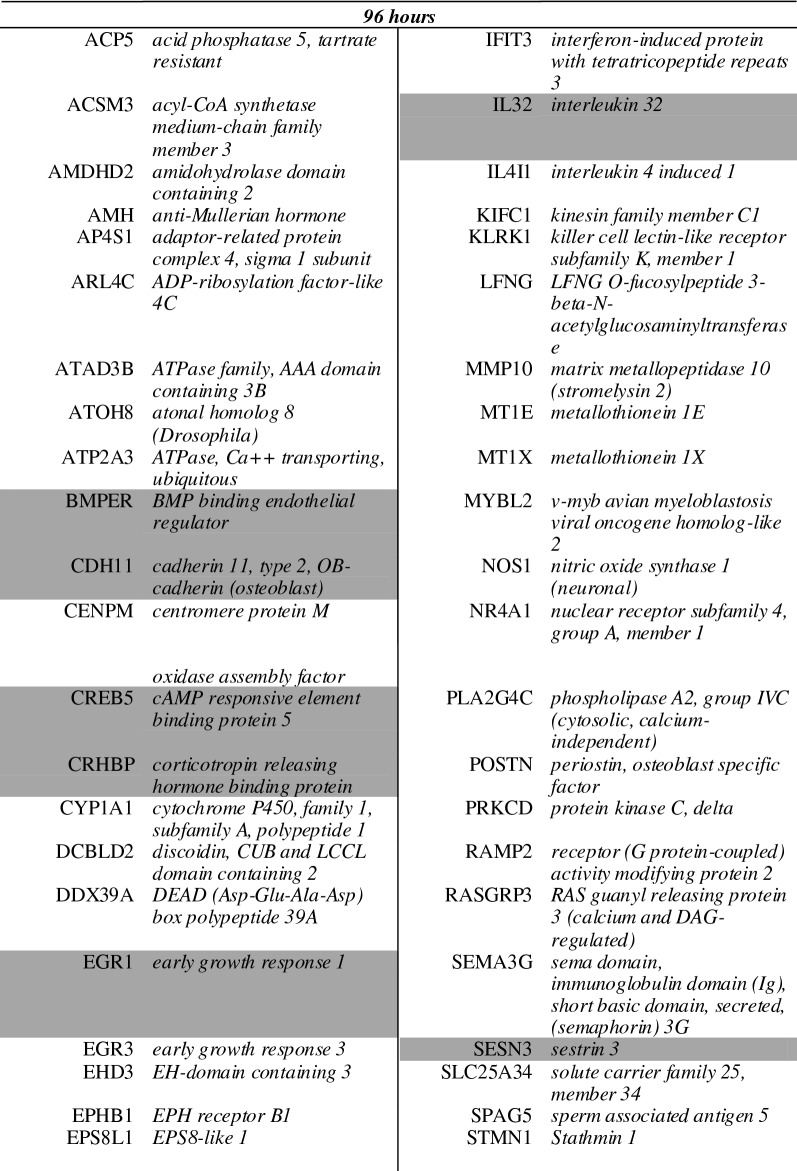

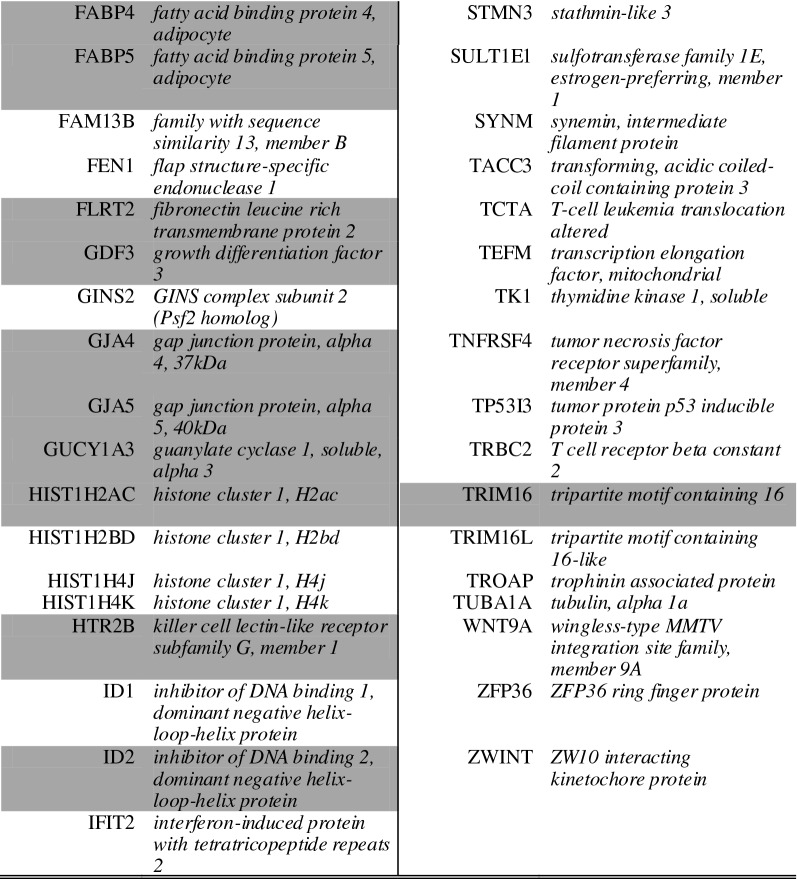
*Overlapping* soloculture enriched genes (ratio ≥ 2) at 24 h (top), 48 h (middle) and 96 h (bottom) identified in both comparisons i.e. soloculture vs. coculture with human pericytes (CHP) and soloculture vs. coculture with bovine pericytes (CBP)

Shading colour highlights soloculture enriched genes that are identified to be overlapping in two (light grey) or all time points (dark grey)

**Table 5:**
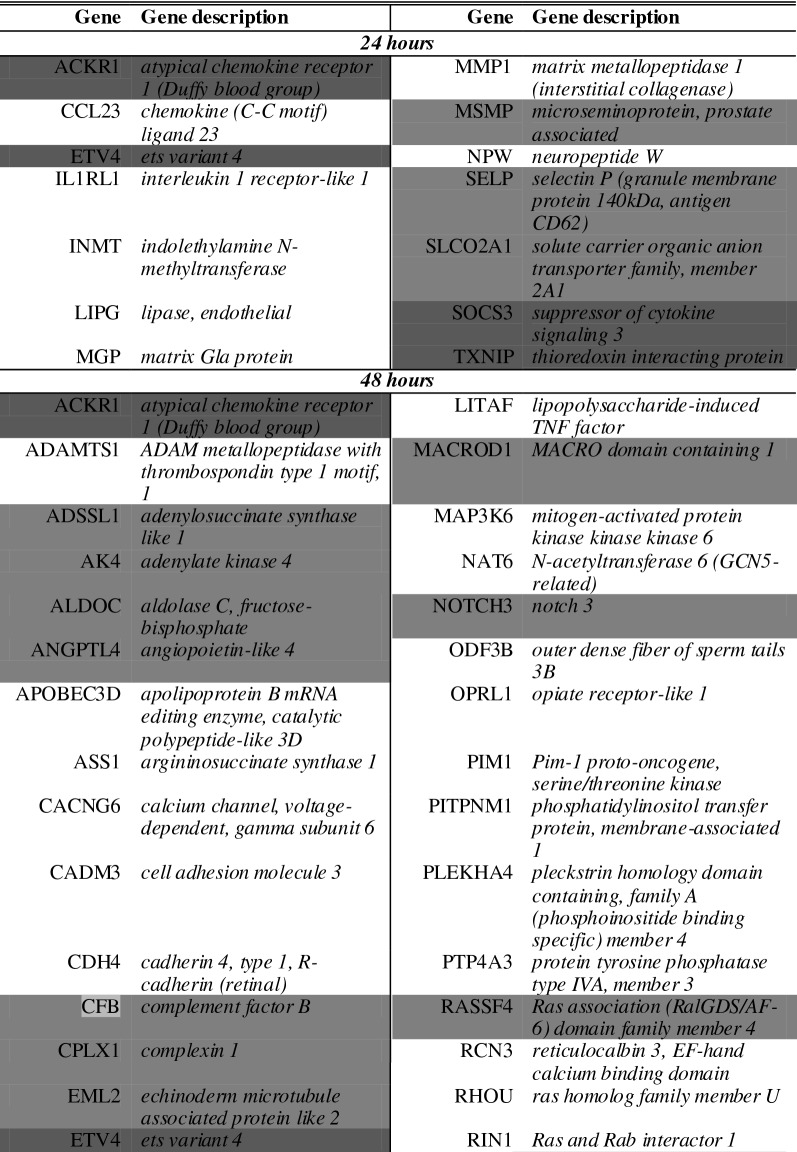

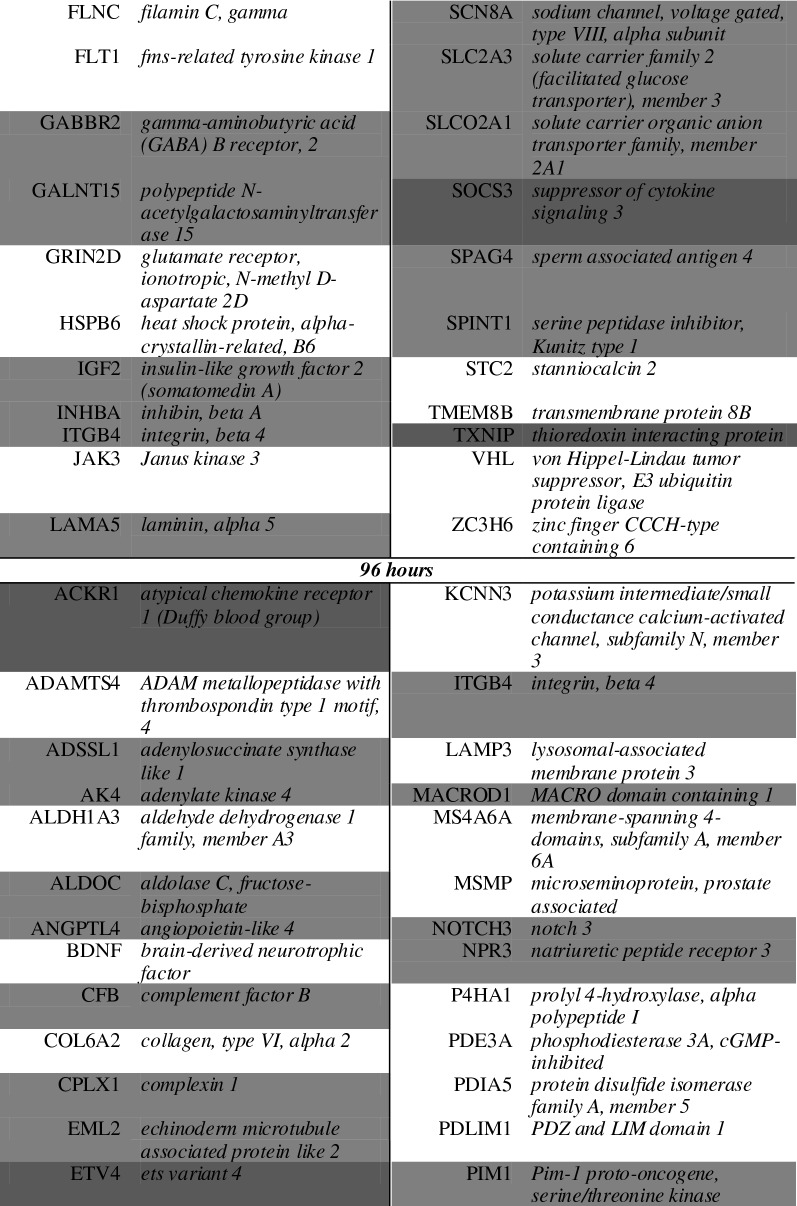

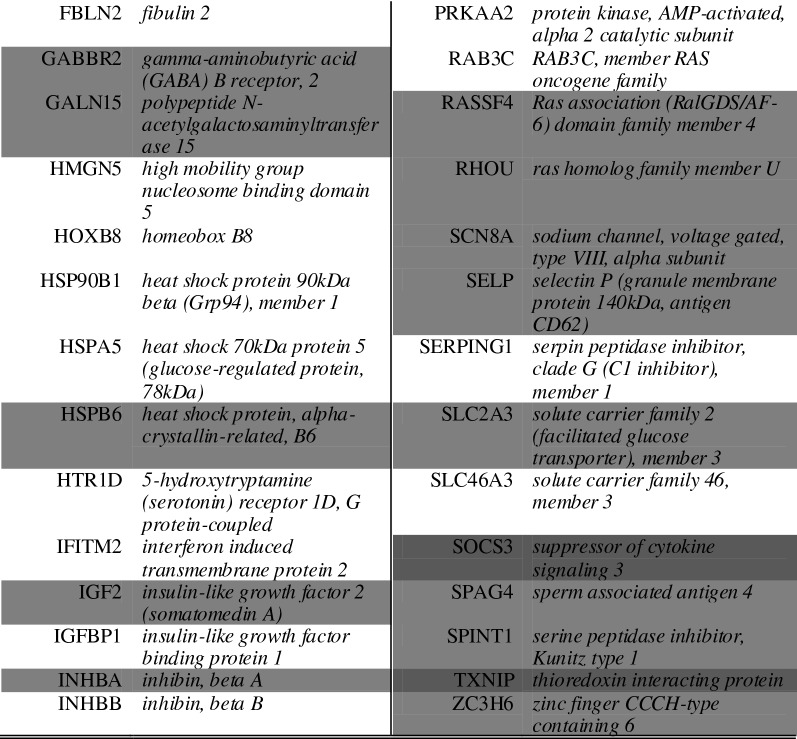
Overlapping coculture enriched genes (ratio ≥ 2) at 24 h (top), 48 h (middle) and 96 h (bottom) identified in both comparisons i.e. soloculture vs. coculture with human pericytes (CHP) and soloculture vs. coculture with bovine pericytes (CBP)

Shading colour highlights coculture enriched genes that are identified to be overlapping in two (light grey) or all time points (dark grey)

#### Gene expression profile of specific genes

**Fig. 5:**
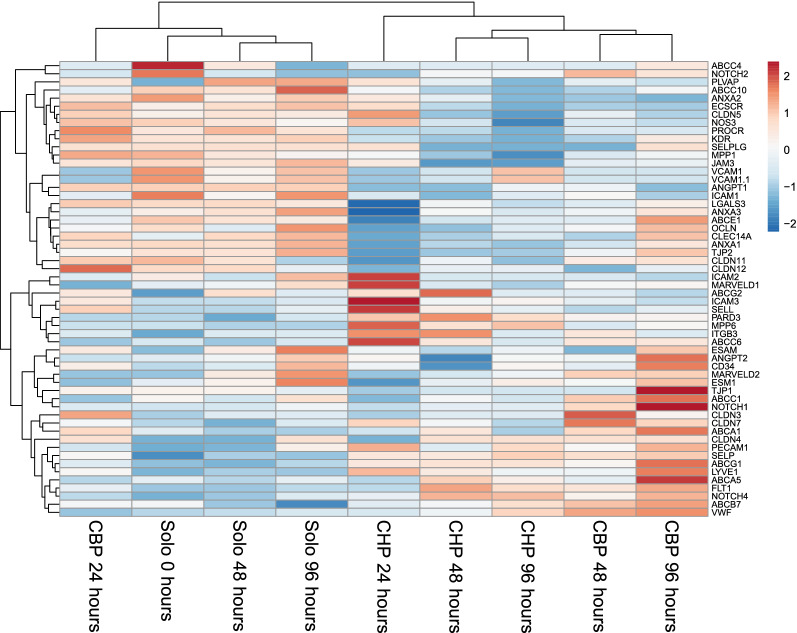
Heat map of vascular genes, junction associated genes, tight junction and tight junction associated genes, ABC transporter genes and endothelial marker genes in endothelial cells. Data input consisted of the normalized expression in solocultured endothelial cells and the normalized expression in cocultured endothelial cells (i.e. with either human pericytes (CHP) or bovine pericytes (CBP). Unit variance scaling is applied to rows

**Fig. 6:**
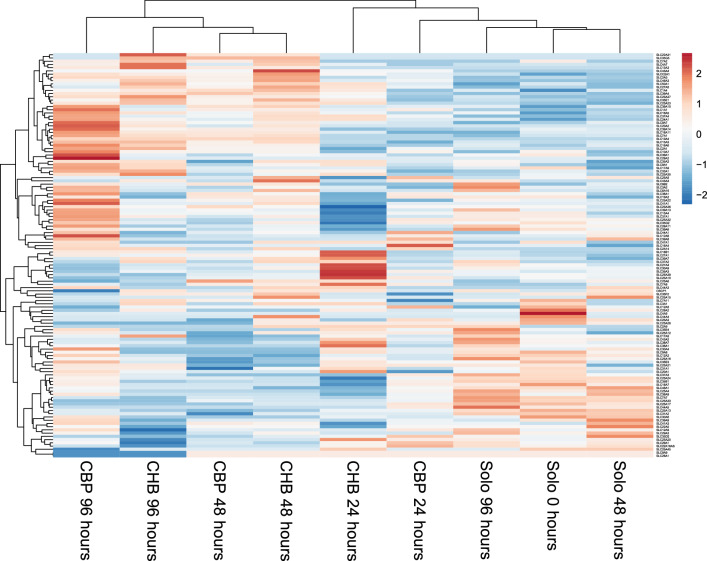
Heatmap of all expressed solute carrier (SLC) transporters. Data input consisted of the normalized expression in solocultured endothelial cells and the normalized expression in cocultured endothelial cells (i.e. with either human pericytes (CHP) or bovine pericytes (CBP). Unit variance scaling is applied to rows

We also assessed the gene expression profile over time for some specific gene groups related to the BBB i.e. vascular permeability genes, junction associated genes, tight junction and tight junction associated genes, ABC transporter genes and endothelial marker genes (Fig. 5), as well as the gene expression profile of solute carrier (SLC) transporter genes (Fig. 6). The latter shows a major clustering in the expression of these genes of both cocultures at 48 h and 96 h in one cluster and soloculture at 0, 48 and 96 h together with both cocultures at 24 h in another cluster. Addition of pericytes is shown to lead to different responses for the different conditions (Figs. 5, 6). Most of these responses were already identified by other studies [6, 10, 11].

Interestingly, among the gene expression profiles of known vascular permeability genes, the plasmalemma vesicle-associated protein (PLVAP) expression is highly increased at 24 h for both cocultures, however it decreases drastically from 24 to 96 h. The expression of the intercellular adhesion molecule 1 (ICAM1) and angiopoietin 1 (ANGPT1) is decreased in both cocultures compared to the soloculture (Fig. 5). The expression profile of several junction associated genes is altered upon the presence of brain pericytes compared to its expression in ECs alone (Fig. 5). And the relative gene expression pattern of several tight junction and tight junction associated genes suggests that important tight junction and tight junction accessory genes are expressed i.e. claudin 3 and 5 (CLDN3, CLDN5), occludin (OCLN), tight junction protein 1 and 2 (TJP1, TJP2) etc. However, their expression is not necessarily increased upon presence of brain pericytes (Fig. 5). The expression pattern of endothelial cell marker genes CD34, C-type lectin domain family 14, member A (CLEC14A), von Willebrand factor (VWF) and nitric oxide synthase (NOS3) at 24 to 96 h shows an increased expression of VWF, a decreased expression of CLEC14A and NOS3 and a steady state for CD34, at least for the coculture with bovine pericytes (Fig. 5). These genes are known endothelial marker genes and are known to be expressed in different type of ECs [6].

#### Influence of pericytes on signaling pathways in brain-like endothelial cells

The GO enrichment analysis identified a number of significantly different gene clusters for soloculture conditions vs. coculture conditions. The number of significant gene clusters per assigned ontology, i.e. molecular function, cellular component and biological process were identified.

Molecular function describes and represents activities rather than entities that occur at the molecular level, such as RNA binding and cytokine binding. A cellular component consists of a component of a cell, that is inherently part of a larger object, such as the ribosome and endoplasmic reticulum. The last ontology parameter represents a biological process and defines a series of events by one or more groups of molecular functions, such as the Wnt signaling pathway and protein folding.

Our results indicate very similar numbers of significant pathways or gene clusters over time for each of the comparisons.

Tables 6 and 7 list the top solo- and coculture enriched pathways per comparison and per time point identified by the GO enrichment tool. Pathways were enriched if p < 0.05 and when the number of significant upregulated genes was at least 1.5 times upregulated in the solo- or coculture condition.

**Table 6:** Top regulated soloculture enriched pathways at 24 h (top), 48 h (middle) and 96 h (bottom) for (A) the comparison of soloculture vs. coculture with human pericytes (CHP); and for (B) the comparison of soloculture vs. coculture with bovine pericytes (CBP)

A.			B.		
24 h			24 h		
Pathway	Ratio	p-value	Pathway	Ratio	p-value
Post-Golgi vesicle mediated transport	1.7	1.10E−06	Regulation of cell morphogenesis involved in differentiation	2.1	6.90E−04
Regulation of programmed cell death	1.7	6.50E−06	Salivary gland morphogenesis	3.0	7.60E−04
Regulation of cell death	1.7	8.70E−06	Salivary gland development	3.0	1.44E−03
Regulation of apoptotic process	1.7	1.40E−05	Lysosomal transport	2.0	1.72E−03
Golgi vesicle transport	1.8	1.90E−05	Establishment of protein localization to plasma membrane	3.3	2.66E−03
Protein folding	1.7	4.10E−05	Protein localization to plasma membrane	2.0	2.94E−03
Regulation of transcription from RNA polymerase II promotor in response to hypoxia	3.8	4.60E−05	*Positive regulation of osteoblast proliferation*	*2.0*	*3.27E*−*03*
Negative regulation of cell death	1.9	5.40E−05	Plasma membrane organization	2.0	4.00E−03
Negative regulation of programmed cell death	1.9	5.70E−05	Toll-like receptor 3 signaling pathway	1.6	5.24E−03
De novo protein folding	2.0	6.60E−05	Exocrine system development	3.0	5.72E−03
48 h			48 h		
*RNA export from nucleus*	*1.5*	*2.30E*−*05*	*RNA splicing, via transesterification reactions*	*4.3*	*9.20E*−*08*
*mRNA export from nucleus*	*1.6*	*7.00E*−*05*	*mRNA splicing, via spliceosome*	*4.1*	*2.70E*−*07*
*Post-Golgi vesicle-mediated transport*	*1.5*	*3.40E*−*04*	*RNA splicing, via transesterification reactions with bulged adenosine*	*4.1*	*2.70E*−*07*
Protein localization to endosome	7.0	5.80E−04	*Regulation of cellular protein catabolic process*	*1.8*	*4.00E*−*07*
Type I interferon production	1.8	7.00E−04	*Regulation of proteolysis involved in cellular protein catabolic process*	*1.9*	*5.80E*−*07*
Regulation of type I interferon production	1.8	7.00E−04	Positive regulation of cellular protein catabolic process	2.5	1.70E−06
Regulation of monocyte differentiation	2.0	7.60E−04	*mRNA metabolic process*	*2.2*	*2.50E*−*06*
Golgi localization	2.0	7.60E−04	*Ribonucleoside triphosphate biosynthetic process*	*1.6*	*2.80E*−*06*
*Protein K48-linked ubiquitination*	*1.6*	*1.00E*−*03*	Positive regulation of proteolysis involved in cellular protein catabolic process	2.7	3.40E−06
*Pyrimidine nucleotide metabolic process*	*1.8*	*1.46E*−*03*	*mRNA processing*	*2.6*	*4.20E*−*06*
96 h			96 h		
Arp2/3 complex-mediated actin nucleation	2.0	7.50E−04	*Positive regulation of protein ubiquitination*	*2.8*	*1.00E*−*05*
Pyrimidine nucleoside triphosphate metabolic process	1.8	1.01E−03	*Positive regulation of ligase activity*	*3.9*	*1.90E*−*05*
Programmed necrotic cell death	4.5	1.88E−03	*Positive regulation of protein modification by small protein conjugation or removal*	*2.6*	*2.00E*−*05*
Necroptotic process	9.0	2.59E−03	Nucleoside monophosphate metabolic process	2.2	2.70E−05
*Sprouting angiogenesis*	*2.0*	*3.53E*−*03*	*Positive regulation of ubiquitin*-*protein transferase activity*	*4.3*	*4.40E*−*05*
Regulation of necroptotic process	5.0	3.74E−03	*Regulation of protein modification by small protein conjugation or removal*	*2.1*	*8.00E*−*05*
Fibroblast apoptotic process	2.0	3.74E−03	*Regulation of protein ubiquitination*	*2.1*	*8.60E*−*05*
*Deoxyribonucleotide biosynthetic process*	*4.0*	*4.17E*−*03*	Ribonucleoside monophosphate metabolic process	1.9	1.10E−04
*Positive regulation of protein catabolic process*	*2.0*	*5.23E*−*03*	Regulation of ligase activity	*4.0*	*2.30E−04*
*Positive regulation of protein modification by small protein conjugation or removal*	*1.8*	*5.81E*−*03*	Positive regulation of execution phase of apoptosis	*4.0*	*3.50E−04*

Pathways in italic are soloculture enriched pathways identified in both comparisons for that specific time point. The 2nd column represents the ratio of upregulated and downregulated genes in the pathway under study (ratio ≥ 1.5)

**Table 7:** Top 10 regulated coculture enriched pathways at 24 h (top), 48 h (middle) and 96 h (bottom) for (A) the comparison of soloculture vs. coculture with human pericytes (CHP); and for (B) the comparison of soloculture vs. coculture with bovine pericytes (CBP)

A.			B.		
24 h			24 h		
Pathway	Ratio	p-value	Pathway	Ratio	p-value
Formation of translation preinitiation complex	0.3	6.80E−05	Regulation of cell division	0.3	1.90E−05
Endoplasmic reticulum calcium ion homeostasis	0.1	1.25E−03	Endothelial cell migration	0.3	2.20E−05
Porphyrin-containing compound biosynthetic process	0.4	1.33E−03	*Sprouting angiogenesis*	*0.4*	*2.80*E−*05*
Ribonucleoprotein complex assembly	0.4	2.31E−03	Protein localization to organelle	0.4	3.00E−05
Tetrapyrrole biosynthetic process	0.4	3.38E−03	Blood vessel endothelial cell migration	0.4	3.50E−05
Cytoskeleton-dependent cytokinesis	0.4	4.09E−03	Chromosome segregation	0.2	4.90E−05
Pigment biosynthetic process	0.5	6.86E−03	Epithelial cell migration	0.5	1.10E−04
Negative regulation of centrosome duplication	0.3	9.16E−03	*Ameboidal-type cell migration*	*0.5*	*1.10E*−*04*
Negative regulation of endoplasmic reticulum calcium ion concentration	0.3	9.16E−03	Viral life cycle	0.4	1.30E−04
Negative regulation of centrosome cycle	0.3	9.16E−03	Tissue migration	0.5	1.30E−04
48 h			48 h		
*Notch signaling pathway*	*0.5*	*4.60E−05*	*Epithelial cell migration*	*0.4*	*3.00E−04*
*Hyaluronan catabolic process*	*0.1*	*4.50E−04*	*Epithelium migration*	*0.4*	*3.70E−04*
*Nuclear-transcribed mRNA poly(A) tail shortening*	*0.4*	*5.90E−04*	*Tissue migration*	*0.4*	*3.70E−04*
Formation of translation preinitiation complex	0.2	9.50E***−***04	*Cell junction assembly*	*0.4*	*4.20E−04*
Response to cold	0.3	2.90E***−***04	*Regulation of cell migration involved in sprouting angiogenesis*	*0.3*	*5.20E−04*
mRNA catabolic process	0.5	3.19E***−***03	*Regulation of sprouting angiogenesis*	*0.3*	*5.20E−04*
Autonomic nervous system development	0.2	4.49E***−***03	*Regulation of blood vessel endothelial cell migration*	*0.2*	*5.30E−04*
*Polysaccharide biosynthetic process*	*0.5*	*5.17E−03*	*Carbohydrate biosynthetic process*	*0.4*	*5.40E−04*
Lipopolysaccharide metabolic process	0.3	8.03E***−***03	*Blood vessel endothelial cell migration*	*0.4*	*5.60E−04*
Lipopolysaccharide biosynthetic process	0.3	8.03E***−***03	*Cell junction organization*	*0.4*	*6.10E−04*
96 h			96 h		
Translational termination	0.0	6.80E***−***24	*Notch signaling pathway*	*0.3*	*8.20E−05*
Nuclear-transcribed mRNA catabolic process	0.1	1.70E***−***22	*Response to endoplasmic reticulum stress*	*0.4*	*8.20E−05*
Cotranslational protein targeting to membrane	0.0	1.60E***−***21	*Peptidyl-proline hydroxylation*	*0.2*	*2.17E−03*
Protein targeting to ER	0.0	3.90E***−***21	*Post-translational protein modification*	*0.3*	*2.17E−03*
SRP-dependent cotranslational protein targeting to membrane	0.0	4.70E***−***21	*Positive regulation of gene expression (epigenetic)*	*0.3*	*2.91E****−****03*
Protein localization to endoplasmic reticulum	0.0	4.80E***−***21	*Regulation of mRNA catabolic process*	*0.3*	*2.95E−03*
Establishment of protein localization to endoplasmic reticulum	0.0	2.10E***−***20	*Maintenance of protein localization in organelle*	*0.1*	*3.35E−03*
Nuclear-transcribed mRNA catabolic process	0.1	4.10E***−***20	*Maintenance of protein localization in endoplasmic reticulum*	*0.0*	*3.35E−03*
mRNA catabolic process	0.1	2.70E***−***19	*Notch signaling involved in heart development*	*0.2*	*3.98E−03*
Translational initiation	0.1	1.00E***−***18	Vesicle targeting to, from or within Golgi	0.0	4.03E***−***03

Pathways in italic are coculture enriched pathways identified in both comparisons for that specific time point. The 2nd column represents the ratio of upregulated and downregulated genes in the pathway under study (ratio ≥ 0.5)

Table 8, 9, 10 and 11 list several top regulated significant pathways or gene clusters per comparison and per time point (i.e. 24 and 96 h) that were identified by using the KOBAS software, thereby focusing on databases like Panther, KEGG and Reactome [37].

**Table 8:** Top differentially expressed pathways for the comparison of soloculture vs. coculture with bovine pericytes (CBP) at 24 h

Term	Database	Corrected p-value
Osteoclast differentiation	KEGG	8.46E−04
Regulation of insulin-like growth factor (IGF) transport and uptake by insulin-like growth factor binding proteins (IGFBPs)	Reactome	0.001
Cytokine Signaling In Immune System	Reactome	0.002
Syndecan interactions	Reactome	0.003
Extracellular matrix organization	Reactome	0.004
TNF signaling pathway	KEGG	0.004
Notch signaling pathway	PANTHER	0.006
Immune system	Reactome	0.006
Cell surface interactions at the vascular wall	Reactome	0.012
Non-integrin membrane-ECM interactions	Reactome	0.014
Interferon alpha/beta signaling	Reactome	0.017
Interferon signaling	Reactome	0.019
Hemostasis	Reactome	0.020
ECM proteoglycans	Reactome	0.022
Platelet degranulation	Reactome	0.022
Response to elevated platelet cytosolic Ca2+	Reactome	0.024
Nicotine degradation	PANTHER	0.028
Molecules associated with elastic fibers	Reactome	0.029
Integrin cell surface interactions	Reactome	0.030
Tryptophan metabolism	KEGG	0.031
Hepatitis B	KEGG	0.032
PECAM1 interactions	Reactome	0.040
Elastic fiber formation	Reactome	0.041
AGE-RAGE signaling pathway in diabetic complications	KEGG	0.046
GRB2/SOS provides linkage to MAPK signaling for Integrins	Reactome	0.047

Pathway analysis was conducted using different databases i.e. KEGG pathway, Reactome and Panther [37]. Statistical analysis was performed using the Fisher’s exact test and false discovery rate correction test was performed by the Benjamini and Hochberg method [3]

**Table 9:** Top differentially expressed pathways for the comparison of soloculture vs. coculture with human pericytes (CHP) at 24 h

Term	Database	Corrected p-value
AGE-RAGE signaling pathway in diabetic complications	KEGG	0.002
Hemostasis	Reactome	0.002
Cell adhesion molecules (CAMs)	KEGG	0.006
Senescence-associated secretory phenotype (SASP)	Reactome	0.007
TNF signaling pathway	KEGG	0.009
CCKR signaling map	PANTHER	0.009
Cell surface interactions at the vascular wall	Reactome	0.014
Chemokine signaling pathway	KEGG	0.016
Osteoclast differentiation	KEGG	0.022
Signaling by cytosolic FGFR1 fusion mutants	Reactome	0.023
Syndecan interactions	Reactome	0.025
Estrogen signaling pathway	KEGG	0.032
Extracellular matrix organization	Reactome	0.035
Propanoate metabolism	KEGG	0.036
2-Oxobutanoate degradation	BioCyc	0.037
Valine, leucine and isoleucine degradation	KEGG	0.038
Integrin cell surface interactions	Reactome	0.040
Immune system	Reactome	0.047

Pathway analysis was conducted using different databases i.e. KEGG pathway, Reactome and Panther. Statistical analysis was performed using the Fisher’s exact test and false discovery rate correction test was performed by the Benjamini and Hochberg method [3]

**Table 10:** Top differentially expressed pathways for the comparison of soloculture vs. coculture with bovine pericytes (CBP) at 96 h

Term	Database	Corrected p-value
Regulation of insulin-like growth factor (IGF) transport and uptake by insulin-like growth factor binding proteins (IGFBPs)	Reactome	3.31E−04
Gap junction assembly	Reactome	0.003
Gap junction trafficking	Reactome	0.008
Gap junction trafficking and regulation	Reactome	0.008
Degradation of the extracellular matrix	Reactome	0.012
Inhibition of voltage gated Ca^2+^ channels via beta/gamma subunits	Reactome	0.015
Activation of G protein gated potassium channels	Reactome	0.015
G protein gated potassium channels	Reactome	0.015
Cell cycle, mitotic	Reactome	0.018
Cell cycle	Reactome	0.020
Extracellular matrix organization	Reactome	0.021
Inwardly rectifying K^+^ channels	Reactome	0.027
Phenylalanine metabolism	KEGG	0.027
Deposition of new CENPA-containing nucleosomes at the centromere	Reactome	0.038
Nucleosome assembly	Reactome	0.038
Intrinsic pathway of fibrin clot formation	Reactome	0.047
GABA B receptor activation	Reactome	0.048
Activation of GABAB receptors	Reactome	0.048

Pathway analysis was conducted using different databases i.e. KEGG pathway, Reactome and Panther. Statistical analysis was performed using the Fisher’s exact test and false discovery rate correction test was performed by the Benjamini and Hochberg method [3]

**Table 11:** Top differentially expressed pathways for the comparison of soloculture vs. coculture with human pericytes (CHP) at 96 h

Term	Database	Corrected p-value
RHO GTPase effectors	Reactome	1.02E−04
DNA methylation	Reactome	0.004
SIRT1 negatively regulates rRNA expression	Reactome	0.005
Activated PKN1 stimulates transcription of AR (androgen receptor) regulated genes KLK2 and KLK3	Reactome	0.005
RHO GTPases activate formins	Reactome	0.008
RNA polymerase I promoter opening	Reactome	0.009
B-WICH complex positively regulates rRNA expression	Reactome	0.010
RHO GTPases activate PKNs	Reactome	0.0131
Signal Transduction	Reactome	0.022
Formation of the beta-catenin/TCF transactivating complex	Reactome	0.024
G1/S-specific transcription	Reactome	0.026
Mineral absorption	KEGG	0.032
Epigenetic regulation of gene expression	Reactome	0.038
Extracellular matrix organization	Reactome	0.041
Regulation of insulin-like growth factor (IGF) transport and uptake by insulin-like growth factor binding proteins (IGFBPs)	Reactome	0.046

Pathway analysis was conducted using different databases i.e. KEGG pathway, Reactome and Panther. Statistical analysis was performed using the Fisher’s exact test and false discovery rate correction test was performed by the Benjamini and Hochberg method [3]

**Fig. 7:**
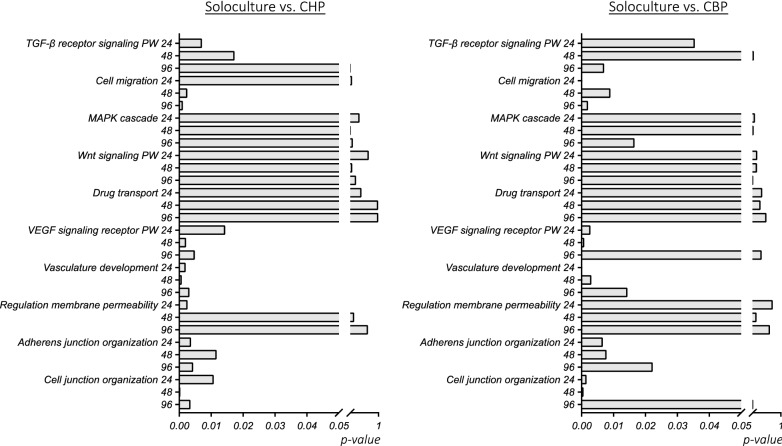
Pathway (PW) analysis of several BBB-related PWs for the comparison of solocultured vs. cocultured endothelial cells with (A) human pericytes (CHP); and with B) bovine pericytes (CBP) at different time points (i.e. 24, 48 and 96 h). The x-axis depicts the p-value. Data analysis was conducted using the GO enrichment tool of GenXPro

Besides a general analysis of the number of differentially expressed pathways, analysis was performed for specific gene clusters or pathways that are known to relate to BBB induction, maturation or maintenance (Fig. 7). Other gene clusters like those for transferrin transport, organic acid transport, anion transport and L-amino acid transport did not show any significant difference between the solo- and coculture (data not shown). Our results show a general high variability in differential expression over time, indicating the importance of time.

An interesting cluster of genes is the one related to the regulation of membrane permeability. The latter is significantly enriched in the soloculture compared to CHP at 24 h, after which significance is lost (at 48 h and 96 h), although the soloculture shows a general upregulation. For the comparison between the soloculture and CBP, this cluster of genes related to the regulation of membrane permeability, is the only cluster of genes in this graph that is enriched for the soloculture. This indicates that the genes that significantly differ are upregulated in the soloculture. The cluster of genes related to the p38/mitogen-activated protein kinase (MAPK) pathway is shown to be differentially expressed only at 48 h after putting ECs into coculture. This pathway is known to promote endothelial cell migration. The gene set related to transforming growth factor (TGF)-b is shown to be differentially expressed for at least some time points in both cocultures.

## Discussion

### Influence of brain pericytes on functional barrier properties: Barrier tightness and efflux transporter functionality

The tightening of junctions between human ECs, resulting in a decreased permeability to non-permeant markers, such as LY, is demonstrated to occur when ECs are cultivated with pericytes of either bovine (i.e. primary cells) or human (i.e. cell line) origin. This confirms the contribution of soluble factors secreted by brain pericytes in ECs barrier tightening. Therefore, this in vitro set-up proved to be suitable for investigation of the molecular mechanisms responsible for establishment of this important BBB feature in human ECs by RNA sequencing.

### Influence of brain pericytes on the transcriptomic profile of brain-like endothelial cells

Some important mechanisms involved in BBB establishment and maturation, notably the influence of brain pericytes, remain poorly understood. For this, we investigated transcriptional changes in ECs in soloculture compared to in ECs in coculture with brain pericytes.

#### Influence of pericytes on the global gene expression profile of brain-like endothelial cells

Compared to the total number of identified genes, only few genes are differentially expressed when comparing solocultured and cocultured ECs. This result suggests that only a small percentage of genes is responsible for the observed barrier tightening in ECs. The number of differentially expressed genes between solo- and coculture is increasing with coculture duration. Indeed, this was expected as the EC monolayer was tighter after 96 h in presence of pericytes than after 24 h.

#### Enriched gene expression

The increasing number of soloculture enriched genes over time, while the expression of those genes is not differentially regulated in the soloculture over time, might actually reflect their downregulation in the CBP and CHP. Indeed, the importance of downregulated genes in ECs, as an integral part of the BBB signature, was already pointed out by Daneman et al. [10] while comparing BBB ECs with peripheral ECs in mice.

Although, some differences can be observed in the transcriptomic profile of the two cocultures, these differences might simply reflect the differences between the two cell types (i.e. cell line for human pericytes vs. primary cells for bovine pericytes). Indeed, this was not the scope of our study. However, the fact that some genes are regulated in both cocultures, while compared to soloculture, reinforces the potential importance of those genes in the barrier tightening that was observed in both cocultures.

Although, the role of those genes should be further evaluated using other techniques, some of them were already found to be relevant regarding the BBB in other studies. As an example, the Duffy antigen receptor (ACKR1) is an interesting gene listed in the top 10 of coculture enriched genes for both the CBP and the CHP at all time points. ACKR1 is a non-specific receptor for several pro-inflammatory chemokines such as the chemokine (C-X-C motif) ligand 2 (CXCL2) [17, 35]. One role of ACKR1 is to retain neutrophil-derived CXCL2 at the endothelial junctions to regulate its unidirectional migration across the venule wall. This has recently been demonstrated by a study of Girbl et al. [17]. However, the reason behind the observed high expression of ACKR1 in cocultured ECs in this study will have to be further evaluated. It is likely that this is related to the secretion of CXCL1 by pericytes (and ECs), which on its turn forms a cue for neutrophils that are known to secrete CXCL2 [17].

Another interesting gene is the bone morphogenetic protein endothelial cell precursor-derived regulator (BMPER) which is indicated as a soloculture enriched gene at several time points. The high expression of BMPER in solocultured ECs might be explained by its relation to forkhead box O 3a (FoxO3a), which is a transcription factor involved in the regulation of endothelial permeability [25].

#### Gene expression profile of specific genes

The expression of BBB-related genes in ECs is not necessarily depicting the main change upon pericyte induction, as for example genes that are known to increase vascular permeability, e.g. ANGPT2, PLVAP and ICAM1 are demonstrated to be upregulated with a loss of pericytes [10, 22]. Our data only partially reflects this as only some of these vascular-important genes are decreased, e.g. PLVAP, for which expression decreases drastically from 24 to 48 h and for ICAM1 and ANGPT1, which are genes known to increase permeability.

Tight junction proteins are important proteins known to regulate BBB permeability thereby restricting the paracellular pathway [13, 19, 20]. Our results show expression of several important tight junction genes and tight junction-associated genes, however, they are not characterized by a drastic response upon presence of brain pericytes, although some show a visible response e.g. CLDN3, CLDN7, CLDN10. This stipulates the hypothesis explained above of Daneman et al. [10] and confirms results from other studies [14].

Our data does therefore not directly lead to a conclusive explanation that clarifies the observed difference in endothelial permeability for cocultured ECs compared to solocultured ECs, which suggests that the presence of pericytes alone does not define the BBB.

#### Influence of pericytes on signaling pathways in brain-like endothelial cells

Although, focusing on individual genes might be an easy way to discover novel genes related to BBB formation and maintenance, it is essential to identify how different genes interact with each other in order to fully understand the dynamic regulation of the BBB [5].

Therefore, pathway analysis is a valuable tool to find groups of functionally important genes [24]. Induction and formation occurs through a multiple-step process comprising a broad variety of signaling pathways, all directed by signals of different cell types. For example, vascular endothelial growth factor (VEGF)-related pathways such as the p38/MAPK pathway, play a key regulative role in proliferation, survival and migration of ECs [30]. Other signaling pathways like TGF-b, Angpt 1 and 2, notch and the phosphoinositide 3-kinase (PI3K) pathway are essential for BBB development and maintenance, by supporting a pericyte function. The latter is demonstrated by studies that show defective BBB formation upon pericyte absence [11, 22, 30].

Our results indicate significant association of multiple pathways expressed in ECs with the introduction of brain pericytes. However, the Wnt pathway, which is one of the most important pathways known to regulate BBB formation, does not show to be differentially expressed in ECs between solo- and cocultures. The Wnt pathway is known to induce BBB features such as the expression of tight junctions and expression of specific transporters (e.g. SLC2A1 or GLUT-1), and central nervous system-specific angiogenesis during embryogenesis [24, 30]. Except for SLC2A1, other Wnt-related genes known to be regulated by b-catenin (e.g. LEF1, APCDD1, AXIN2, STRA6, SLC2A1) are, at the contrary, only poorly expressed in our study [30]. Nevertheless, these results might just indicate an earlier time point of activation of this pathway than the time points considered within this study. This would be in line with the fact that induction of BBB properties is initiated by Wnt activation by neural precursors [5]. Considering the previous, we should be aware of the presence of other important pathways and signaling cascades that take place at an earlier time point than the first analysed time point in this study (i.e. 24 h).

Although, some important pathways were discovered over the years, many of the molecular mechanisms behind pericyte-endothelial interactions or behind BBB formation and maintenance are yet to be discovered. The results obtained in this study might be used in further analysis.

## Conclusion

BBB formation and maintenance are complexly regulated by several pathways and signaling cascades, which are activated by different kind of signals. The processes are known to be regulated in a spatial–temporal manner and involve the interaction of different cell types of the NVU and brain ECs [5]. Human in vivo assessment of the molecular mechanisms behind BBB formation and maintenance is hampered by ethical and practical issues. However, well-designed in vitro models that use human cells from a different origin (e.g. stem cells) can greatly benefit this research because of their non-invasiveness and ‘easy-to-handle’-characteristics. They also circumvent the many obstacles related to the use of primary human brain cells which is evidenced by the cumbersome extraction of pure brain capillary ECs out of human brain tissue [24]. Although, advancements over the last years in purification techniques (e.g. fluorescence-activated cell sorting and magnetic bead immunoprecipitation) do enhance research possibilities, and in vitro models using human stem cells are still beneficial as availability of healthy human brain tissue is scarce. In vitro models allow to focus on the interaction between ECs and other cell types of the NVU, thereby decomposing the contribution of different elements of the NVU. Of course, it should be emphasized that this type of studies does not represent the whole BBB physiology, as it lacks several components like the extracellular matrix or other cell types of the NVU, however, the use of these in vitro BBB models in RNA sequencing analysis can reveal enriched genes and pathways that are involved in the response of ECs to paracrine signals delivered by brain pericytes. Besides lacking physiological parameters, sequencing of additional samples would ameliorate the statistical power for identification of significantly differentially expressed genes. Unlike qRT-PCR, RNA sequencing allows the evaluation of all potential important genes as the method does not rely on the use of specific probes for detection of initially chosen genes [34].

This study therefore performed RNA sequencing (MACE) of ECs derived from cord-blood hematopoietic stem cells that were cultured in absence (i.e. soloculture) or presence (i.e. coculture) of brain pericytes. Comparison of both transcriptomes resulted in the identification of a set of upregulated genes in soloculture conditions (soloculture enriched genes) and a set of upregulated genes in coculture conditions (coculture enriched genes). Thereby, several typical BBB genes showed an upregulation, as well as several typical vascular genes showed a downregulation in coculture conditions.

Besides transcriptomics, all other types of ‘omics’ (e.g. proteomics, metabolomics and lipidomics) can be complementary used to provide crucial details on the actual output of these enriched genes as it is known that transcript abundance show low correspondence to protein level [24]. Integration of these different types of datasets can result in a more comprehensive image of the BBB vasculature [24, 38]. Therefore, the transcriptomic datasets that resulted from this study provide a starting point to discover the mechanisms behind BBB formation and maintenance for further validation of interesting soloculture and coculture enriched genes or for micro RNA (miRNA) analysis. miRNAs are known to be capable of individually regulating many mRNA transcripts by mRNA degradation or inhibition of protein translation [31]. Several studies thereby evidence an important role of endogenous miRNAs in the regulation of BBB function [26]. Preliminary evaluation of miRNA expression, for which data is not shown, resulted in the identification of some specific significantly altered miRNAs. Analysis of target genes regulated by these miRNAs subsequently identified a number solo- or coculture enriched transcripts, which suggests importance of miRNA in the regulation of enriched genes. Further analysis, that links dysregulated miRNA expression to differentially expressed genes that are likely related to BBB formation and maintenance, might therefore be essential to provide a complete understanding of the regulation of important BBB genes.

The number of transcriptomic profiling studies of the endothelial barriers, including the BBB, and vasculature in general, did drastically increase over the last years. The latter resulted in the development of transcriptomic databases [38]. The power of these databases is underestimated as they bear a massive amount of valuable data. Although comparative studies of transcriptomic data can be trivial, it may further delineate the molecular mechanisms behind BBB formation and BBB maintenance.

Within the framework of the European Brain Barriers Training Network (H2020-MSCA-ITN-2015), called BtRAIN, a BBBHub (Interfaculty Bioinformatics Unit, UBern, Switzerland) is currently being developed in order to collect and disseminate transcriptomic (resulting from RNA sequencing) data from a variety of studies (in vitro and in vivo, across different species). The transcriptomic data generated in this study will be available in this BBBHub (http://bbbhub.unibe.ch/) upon launch which will allow further use of this data in comparative cross-species and cross-system analysis, due to homogeny of the data analysis process and the presence of substantial metadata. This will further validate the data obtained in this study. For example, a drawback of the present study is the sole analysis of the BBB in static conditions, as the experimental design is lacking physiological parameters such as mechanical forces (e.g. shear stress). The latter is demonstrated to increase the ECs expression of cytoskeletal genes [24]. It would therefore be valuable to compare genes that were found to be enriched in the present study to the transcriptomic profile of cocultured ECs in dynamic, flow conditions. However, caution should be made when comparing datasets from different studies as different experimental conditions with different variables pose a challenge for generating conclusive interpretations e.g. inter-individual and regional variability [4, 38].

## Supplementary information

**Additional file 1.** Supplementary table depicting sequencing statistics. Number of raw reads sequenced and number of unique reads after removal of duplicates generated during polymerase chain reaction.

## Abbreviations

ABC: ATP-binding cassette
BBB: Blood–brain barrier
BCRP: Breast cancer resistance protein
CHP: Coculture with human pericytes
CBP: Coculture with bovine pericytes
EC: Endtohelial cells
GF: Elacridar
GO: Gene ontology
LY: Lucifer yellow
MACE: Massive Analysis of cDNA Ends
miRNA: micro RNA
NVU: Neurovascular unit
Pe: Permeability coefficient
P-gp: *P*-Glycoprotein
R123: Rhodamine 123
RH: Krebs–Ringer HEPES buffer

## Footnotes

1 hg38, http://genome.ucsc.edu/cgi-bin/hgTables.
