# Supplementary material for: Contribution of brain pericytes in blood–brain barrier formation and maintenance: a transcriptomic study of cocultured human endothelial cells derived from hematopoietic stem cells
Source: Fluids Barriers CNS. 2020 Jul 28;17:48. doi: 10.1186/s12987-020-00208-1 (PMC7385894; doi:10.1186/s12987-020-00208-1)
Supplement: Supplementary file 1 — Additional file 1. Supplementary table depicting sequencing statistics. Number of raw reads sequenced and number of unique reads after removal of duplicates generated during polymerase chain reaction. [file 12987_2020_208_MOESM1_ESM.docx]

Additional file 1

TableS1 depicting sequencing statistics. Number of raw reads sequenced and number of unique reads after removal of duplicates generated during polymerase chain reaction.

| **Sample** | **Number of raw sequenced reads** | **Number of reads after PCR duplicate cleaning** |
| --- | --- | --- |
| S-t0-1 | 10265768 | 5567082 |
| S-t0-2 | 10567460 | 6051331 |
| S-t0-3 | 9735964 | 5535192 |
| S-t48-1 | 9366344 | 5464231 |
| S-t48-2 | 9205876 | 5280722 |
| S-t48-3 | 9932302 | 5600199 |
| S-t96-1 | 14495965 | 8167857 |
| S-t96-2 | 13311632 | 7584567 |
| S-t96-3 | 13283125 | 7263912 |
| B-t24-1 | 23196511 | 10095987 |
| B-t24-2 | 16693965 | 7390992 |
| B-t24-3 | 16975835 | 4518727 |
| B-t48-1 | 20311215 | 6092339 |
| B-t48-2 | 29477370 | 9735967 |
| B-t48-3 | 18028246 | 5617794 |
| B-t96-1 | 18627797 | 8035911 |
| B-t96-2 | 19463083 | 8461272 |
| B-t96-3 | 12362170 | 5390554 |
| H-t24-1 | 14928638 | 5745556 |
| H-t24-2 | 12523844 | 5930228 |
| H-t24-3 | 16571616 | 5925180 |
| H-t48-1 | 10812298 | 5177127 |
| H-t48-2 | 11829783 | 5629142 |
| H-t48-3 | 12684418 | 5003500 |
| H-t96-1 | 12591438 | 5816774 |
| H-t96-2 | 13012807 | 6231959 |
| H-t96-3 | 11362330 | 5131495 |
